# Automating the Calibration of Visible Light Positioning Systems

**DOI:** 10.3390/s22030998

**Published:** 2022-01-27

**Authors:** Robin Amsters, Simone Ruberto, Eric Demeester, Nobby Stevens, Peter Slaets

**Affiliations:** 1 Department of Mechanical Engineering, KU Leuven, 3000 Leuven, Belgium; Eric.demeester@kuleuven.be (E.D.); peter.slaets@kuleuven.be (P.S.); Simone.ruberto@student.kuleuven.be (S.R.); 2 Department of Electrical Engineering, KU Leuven, 3000 Leuven, Belgium; nobby.stevens@kuleuven.be

**Keywords:** indoor positioning, visible light positioning, mobile robot, calibration

## Abstract

Visible light positioning is one of the most popular technologies used for indoor positioning research. Like many other technologies, a calibration procedure is required before the system can be used. More specifically, the location and identity of each light source need to be determined. These parameters are often measured manually, which can be a labour-intensive and error-prone process. Previous work proposed the use of a mobile robot for data collection. However, this robot still needed to be steered by a human operator. In this work, we significantly improve the efficiency of calibration by proposing two novel methods that allow the robot to autonomously collect the required calibration data. In postprocessing, the necessary system parameters can be calculated from these data. The first novel method will be referred to as semi-autonomous calibration, and requires some prior knowledge of the LED locations and a map of the environment. The second, fully-autonomous calibration procedure requires no prior knowledge. Simulation results show that the two novel methods are both more accurate than manual steering. Fully autonomous calibration requires approximately the same amount of time to complete, whereas semi-autonomous calibration is significantly faster.

## 1. Introduction

Indoor positioning is a surprisingly large research area. Motivated by the large amount of possible applications, many researchers have developed systems capable of obtaining location data indoors. We refer to these systems as Indoor Positioning Systems (IPS). Many IPS rely on different types of Radio Frequency (RF) signals such as UltraWide Band (UWB) [1], BlueTooth (BT) [2] or Wi-Fi [3]. Based on the signal strength or travel time, the transmitter-receiver distance can be calculated. The receiver location can then be determined through trilateration [4]. Alternatively, fingerprinting techniques are also often used for indoor positioning. Such systems operate in two distinct stages. In the offline stage, a signal strength database is constructed by measuring various wireless signals at known locations. In the second (online) stage, signal fingerprints are matched to the database to obtain the current (unknown) receiver location [4].

In recent years, a new positioning technology is receiving an increasing amount of attention. In Visible Light Positioning (VLP), the intensity of indoor light sources are modulated at rates imperceptible to the human eye. Typically, the lights are continuously transmitting a unique code or frequency [5] that identifies them. Alternatively, they can continuously broadcast their own location [6]. Both photodiodes and cameras can receive the optical signals [6]. Similar to IPS based on RF, both trilateration and fingerprinting techniques can determine the receiver location.

According to a recent survey, VLP is the second most popular positioning technology in indoor positioning research [7]. Only Wi-Fi positioning is used more often [7]. Like many other positioning technologies, VLP systems generally require prior knowledge of certain environmental parameters. In case of trilateration for example, the coordinates of the transmitters need to be known. Fingerprinting techniques rely the previously built signal strength database. These parameters are generally determined through a calibration procedure.

Even though calibration is a prerequisite for positioning, the topic is not often discussed in literature. The necessary data are often obtained through manual measurements, which can be a lengthy and labor-intensive process. Some researchers have attempted to simplify the calibration of fingerprinting systems that use Wi-Fi or other opportunistic signals. However, despite the popularity of visible light positioning, calibration of these systems is rarely discussed. In our previous work [8,9], we proposed the use of a mobile robot to collect the necessary calibration data for a VLP system. In postprocessing, these data could be used to reconstruct a map of the environment with the light source locations and their identifiers. However, the previously proposed procedure still needs a human operator to steer the robot through the environment. This paper will propose two methods by which the robot can gather the calibration data autonomously. In case a map of the building is available, as well as the approximate locations of the lights sources, the robot can plan a path through the environment and measure the light locations and their identifiers accurately. Additionally, this paper also proposes a method that requires no prior knowledge. Instead, the robot autonomously explores the environment and gathers the data. These two novel approaches are compared to manual steering and evaluated through computer simulations. The main contribution of this work lies in automating the data gathering, which can otherwise be very time-consuming. In this paper, the method is applied to the case of visible light positioning. Regardless, the methods can be used for other positioning technologies (such as UWB or WiFi) and techniques (such as fingerprinting) as well.

The rest of this paper is structured as follows: Section 2 discusses related work. Section 3 will describe the simulation environment, the calibration algorithms and the data processing. Simulation results are presented in Section 4 and discussed in Section 5. Finally, a conclusion is drawn in Section 6.

## 2. Related Work

As mentioned in Section 1, the parameters that need to be calibrated depend on the positioning technique and hence influence the calibration of these systems. For an in-depth overview of calibration methods suited for different types of positioning techniques, the reader is referred to our previous work [9]. However, the main distinction in calibration methods is between the calibration of range-based and fingerprinting systems.

Some range-based systems can be calibrated without human effort. These systems typically translate the signal strength or travel time to a transmitter-receiver distance. Each such distance defines a circle along which the receiver can be located. The intersection of three such circles leads to a unique location estimate in 2D [4]. If the transmitters are able to communicate with each other, the same principle can be used for calibration. The signals are then used to determine the approximate distance between transmitters. Given a sufficient number of measurements, the location of each transmitter can be recovered [10]. However, this approach requires that the transmitters can communicate with each other, which is not the case with VLP systems. Most VLP transmitters are simply modulated light sources, and do not have the required hardware to receive signals.

Previous research on the calibration of indoor positioning systems has more often focused on those that use fingerprinting techniques. Constructing the fingerprint database can take a long time, so it is logical to automate this process as much as possible. Some authors proposed crowdsourcing as a method of data gathering [11,12,13,14]. Rather than a dedicated site-survey, the calibration data are gathered by the users of the system. As more data enters the system, the database becomes more complete and therefore more accurate. However, crowdsourcing has lower accuracy, which can be improved to some extent by collecting more data. Nevertheless, the quality of crowdsourced data is generally lower, therefore accuracy is lower compared to other methods. Additionally, crowdsourcing requires users to give up personal data. Even though data can be anonymized, not all users may wish to share it.

A completely different approach relies on mobile robots for data collection. For example, Hähnel et al. [15] used a mobile robot to determine the location of RFID tags in the environment. In a later stage, this information can be used for localization. However, the robot needed to be manually steered through the environment. In contrast, the robots used by [16,17,18] are able to autonomously construct a Wi-Fi fingerprint database. However, the navigation approaches used in these works were relatively simple (e.g., by following a wall as much as possible). Therefore, the calibration may take significantly longer compared to a human operator. Nevertheless, they do succeed in exploring the entire indoor space.

More sophisticated algorithms have been developed that enable mobile robots to autonomously map an indoor space. An often-used method is frontier exploration, which moves the robot to the boundaries between open space, and unknown areas [19]. These boundaries are referred to as frontiers. As the robot moves towards a particular frontier, new information is obtained and the frontier may be moved to the new boundary of known space. Alternatively, the new information may reveal an obstacle that blocks the robot (e.g., a wall). In that case, the frontier can be removed. The robot will continue to explore the environment until there are no frontiers left. Frontier exploration is the most popular algorithm for mobile robot exploration [20]. However, other exploration methods such as those based on Rapidly-exploring Random Trees (RRTs) [21] also exist.

Calibration of VLP systems is seldom discussed in literature. Most works that mention it either calibrate the channel model [22,23], or parameters internal to the receiver [24,25,26]. While this information is needed for PD-based systems, it is not sufficient. Very few publications discuss the calibration of transmitter locations or the fingerprint database of VLP systems, but some do exist. For example, Yue et al. [27] used a smartphone to calibrate a system based on fingerprinting. The trajectory is estimated with Pedestrian Dead Reckoning (PDR) and light intensity. Absolute location fixes are obtained by detecting doors with changes in light intensity and magnetic field strength. Similarly, Liang and Liu [28] used a smartphone to calibrate the location of unmodulated LEDs by combining PDR with opportunistic signals. Both [27,28] require a human surveyor. Additionally, the resulting positioning accuracy is relatively low (0.8 m and 2.30 m on average, respectively). Our previous works [8,9] showed that calibration (and subsequent positioning) accuracy can be very high if a mobile robot is used for calibration. However, those works also required a human operator to steer the robot. In contrast, Glass et al. [29] used an autonomous mobile robot to construct the fingerprint database for a VLP system. Besides a photodiode, the robot was also equipped with a tracker for the HTC VIVE virtual reality system. This tracker allows the location of the robot to be determined very accurately, thereby increasing the accuracy of the fingerprint database. However, similar to [16,17,18], the navigation algorithm was relatively simple. The robot covers the whole space in a zigzag pattern, and needs to stop to record each fingerprint. Additionally, the HTC VIVE can only cover a relatively small space. Therefore, the system needs to be frequently moved in order to calibrate the entire indoor space. Hence, calibration with this method can take a relatively long time.

## 3. Materials and Methods

### 3.1. Simulation Environment

Computer simulations will be used to evaluate the calibration methods proposed in this work. Simulations are an appropriate evaluation tool in this case as we are mostly interested in the relative performance of each method. The accuracy of robot calibration with manual steering was already evaluated in [8,9]. This work will use the same manual calibration method as a point of reference for the automated methods. Additionally, simulations enable much larger experimental spaces. Glass et al. [29] pointed out that current VLP research is often conducted in relatively small spaces.

The open source Gazebo simulator [30] was used to simulate the robot and the environment. Gazebo can be used for the 3D simulation of robots and environments. It can also easily be integrated with the Robot Operating System (ROS), which is an open source framework that is popular in robotics research. ROS provides a standard communication interface in the form of a publish/subscribe architecture. Over the years, many software packages for various purposes have been made available by the community. The large existing codebase makes ROS very attractive to use, as developers have to spend less time “reinventing the wheel” and can instead focus on higher level functionality.

**Figure 1:**
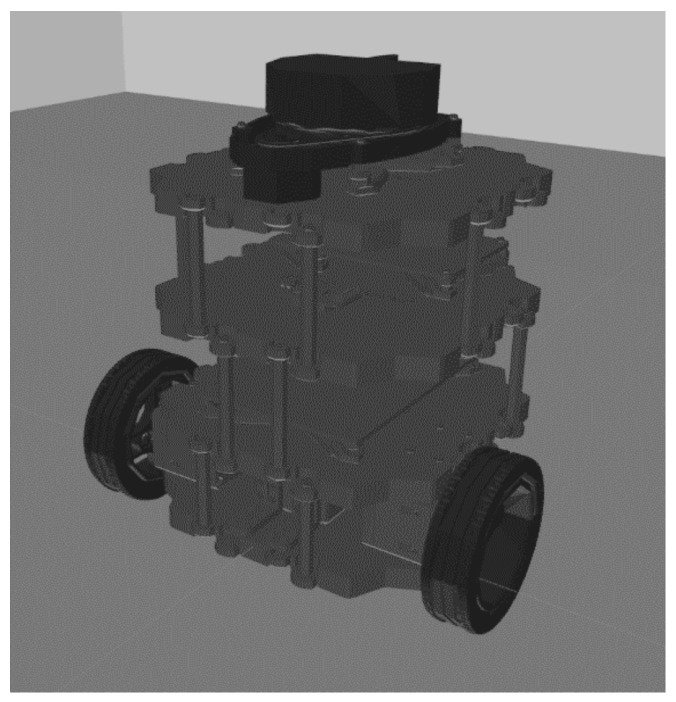
Simulated mobile robot.

A modified Turtlebot 3 was used as a simulated mobile robot (see Figure 1). This robot is equipped with multiple sensors. A 2D LIght Detection And Ranging (LIDAR) sensor is mounted on top of the robot platform and provides environmental perception. Encoders and a gyroscope together provide odometry data. Finally, a ceiling-facing camera was added to detect and calibrate nearby light sources.

VLP systems generally use modulated light sources. Every light continuously emits a unique code or frequency, by which it can be identified. It may be possible to simulate this high-frequency modulation by making a plugin for the Gazebo simulator. However, the focus of this paper is automating the data-collection process, not necessarily the processing of that data. Therefore, our simulations will use Aruco (AR) tags rather than modulated lights. As will be discussed in Section 3.2, this substitution will have little impact on how the data should be interpreted. So long as the image processing pipeline returns an identifier and pixel coordinates, it can be used by the mapping algorithm. This work is mainly interested in the relative performance of the different calibration methods (Section 3.2). Absolute accuracy is less important. Therefore, AR tags can be used as a substitution for LED lights, similar to [31].

In our simulations, the ar-track-alvar package [32] was used to detect the aruco markers. This ROS node processes the images from the camera and returns both the tag ID and its coordinates in the camera frame. This is almost the same output as the image processing pipeline of [8,9]. The only difference is that previous work identified the light by its frequency, whereas the AR tags use an ID.

**Figure 2:**
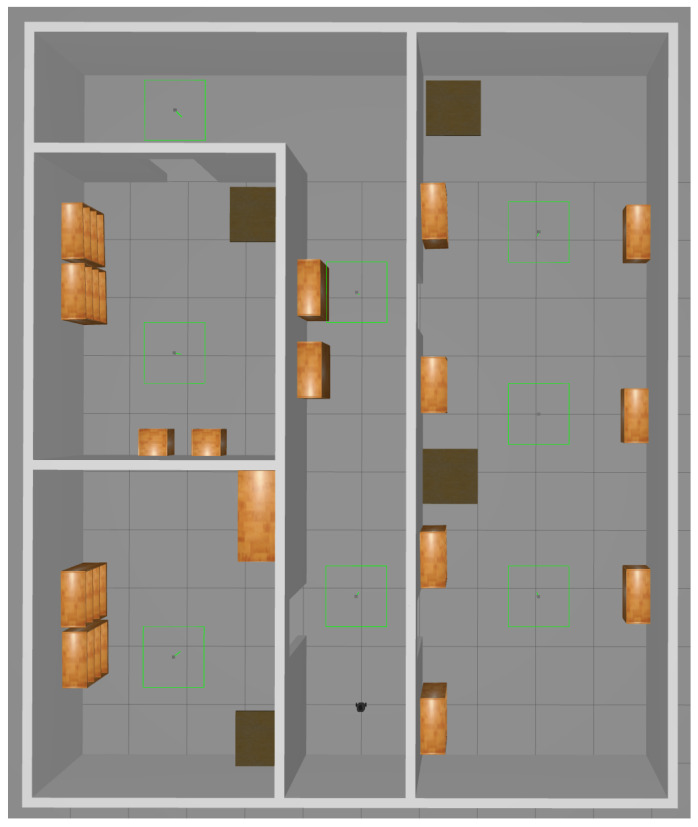
Simulation environment.

The robot will calibrate a simulated environment of approximately 12 m by 10 m (see Figure 2). The environment contains multiple separate rooms and static obstacles that the robot must be able to navigate. The room size and the measurement range are related. Larger rooms will generally have larger open areas. In those cases, a larger measurement range is preferable, otherwise the LIDAR data may have too few features, and the SLAM accuracy (and consequently also the calibration accuracy) decreases. Additionally, a larger measurement range means can result in a shorter calibration time. The sensor can then detect the edges the space from further away, and the distance that the robot has to travel decreases. Our simulation environment has relatively small spaces, hence a relatively short range can be used. In larger, more open spaces such as warehouses, a larger measurement range should be used. The impact of the measurement range is discussed in more detail in Section 3.2.3.

Eight AR tags with unique IDs are present in the environment. Increasing or decreasing the number of tags generally has little impact on the accuracy. The accuracy with which the tags can be positioned is mostly determined by the accuracy of the robot positioning (i.e. the accuracy of the SLAM algorithm) and the image processing pipeline. Changing the number or distribution of the tags will therefore not impact the accuracy. One exception to this is when new tags are located close to an obstacle (such as a chair or table). In that case, the robot will be unable to position the camera directly underneath the tag, and the residual lens distortion may negatively impact performance. However, changes in sensor noise or the available number of features do have a significant impact on the calibration accuracy.

While changing the number of tags does not impact accuracy, it will influence the calibration time. More tags will lead to a longer calibration time and vice versa, as the robot has to stop more or less often to calibrate the tags.

### 3.2. Calibration Methods

In this work, three different calibration methods will be compared, each will be elaborated further in the next sections:

- Manual calibration (Section 3.2.1)
- Semi-autonomous calibration (Section 3.2.2)
- Fully autonomous calibration (Section 3.2.3)

All methods use a mobile robot for data collection, similar to [8,9]. It is important to note that these methods only define the trajectory that the robot will follow. The light detection and data processing are the same for each method. However, ref. [9] showed that the trajectory can have a significant impact on the calibration accuracy. The different methods may therefore differ in their performance. The main calibration parameters per method are collected in Table 1.

During each simulation, data are recorded and processed offline. Specifically, measurements are recorded from the LIDAR and odometry sensors, as well as the velocity commands. The camera images could be recorded as well, but this would result in very large data files. Instead, only the output of the ar_track_alvar node is recorded (namely the tag ID and its coordinates). While it would be possible to process the calibration data online, performing the processing offline enables a better optimization of the followed trajectory through backpropagation. The cartographer algorithm can perform this backpropagation [33], therefore postprocessing can improve calibration accuracy in this case.

The goal of the calibration is to obtain parameters that will be used for positioning in a later stage, hence there is currently little to gain from online processing. Future work could investigate an online procedure that also estimates the uncertainty of the calibrated parameters. The trajectory could then be adjusted to gather more data about uncertain parameters.

**Figure 3:**
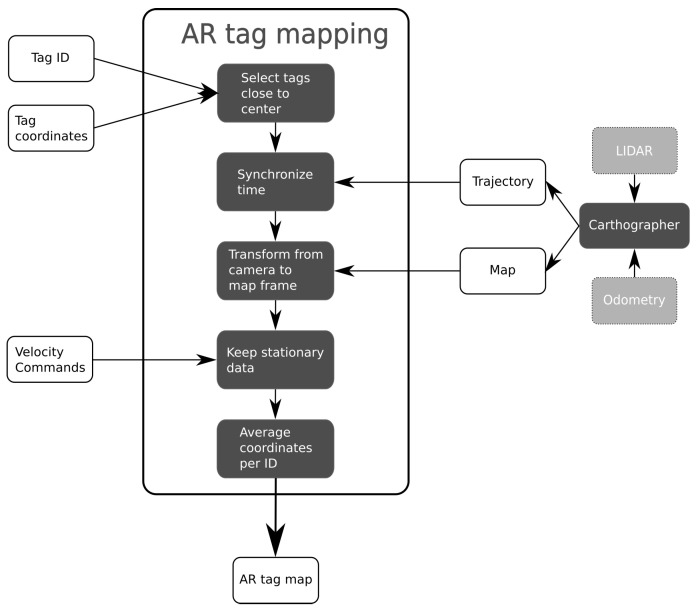
Calibration postprocessing overview.

The postprocessing workflow is illustrated in Figure 3.

After an experiment has concluded, the recorded data are processed very similar to [9]. Tags that were detected too far from the image center, were removed. At the edges of the image, lens distortion is greatest. By only taking tags detected near the center, accuracy can be improved. Simultaneous Localization And Mapping (SLAM) is then used to obtain a map of the environment and the trajectory followed by the robot, based on the LIDAR and odometry measurements. The google cartographer algorithm [33] is used for this purpose. After obtaining the AR tag and SLAM results, both sets of data are time-synchronized. When the timestamps are within 0.1 s of each other, the data are kept. Next, the image coordinates are transformed from the camera frame to the map frame. The position of the camera relative to the robot center is first used to transform the coordinates from the camera to the robot frame. This relative position is fixed and known. Subsequently, the coordinates are transformed from the robot frame to the map frame by using the SLAM trajectory.

Our previous work [9] showed that measurements should be recorded while the robot remains stationary, otherwhise accuracy can be negatively impacted. Therefore, each of the calibration algorithms described in the following sections will make sure that the robot stops moving for a few seconds underneath each AR tag. While the robot is stationary, it is continuously receiving zero velocity commands. The next step in the mapping process therefore checks whether the detected AR tags that remain were measured while the robot was receiving such commands. At this stage, the locations of the AR tags are not a single point, but rather a small cluster. Finally, the position of each tag is obtained as the average of all coordinates of that ID. The end result is a map of the environment with the detected AR tag locations. Examples such a maps can be found in Section 4.

#### 3.2.1. Manual Calibration

**Figure 4:**
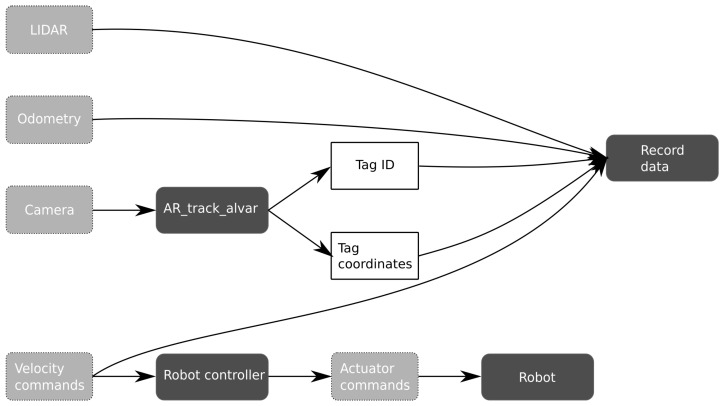
Overview of data flow during manual calibration.

Contrary to Section 1, manual calibration will no longer refer to measurements that have to be collected by hand. Instead, it will refer to how the robot is steered through the environment. In this method, a human operator steers the robot with a remote control. The velocity commands are translated into actuator commands for the motors by the robot controller. At each light source, the robot is stopped and data are collected for a few seconds. Therefore, the human operator takes care of localization and goal selection. This method was initially proposed in [8] and improved in [9]. Figure 4 shows the flow of data during manual calibration.

#### 3.2.2. Semi-Autonomous Calibration

Semi-autonomous calibration relies on the ROS navigation stack [34], which is a collection of software packages that together enable autonomous mobile robot navigation. The Turtlebot 3 comes with all the necessary drivers and configuration files for the navigation stack, and is therefore relatively easy to set up. The navigation stack consists of a few main components:

- The localizationcomponent estimates the current robot position based on the previous position, measurements from the LIDAR (which are matched to the existing map) and odometry data. An Adaptive Monte Carlo Localization (AMCL) [35] is used as the localization algorithm.
- The environment is represented as a costmap, which is a 2D grid of cells that can each be marked as unknown, free space, occupied, or have a cost value that depends on the distance to a certain obstacle [36]. The global costmap is calculated by inflating the obstacles based on the radius of the robot and an optional safety margin. This inflation ensures that even if the path planner calculates a trajectory close to an obstacle, it can still safely be executed [37].
- The local costmap is very similar to the global costmap. However, the local costmap is a scrolling window around the robot, and is continuously updated based on LIDAR data [38]. Therefore, the local costmap also contains moving and previously unknown obstacles.
- The global planner takes as an input the initial robot pose, the desired goal pose and the global costmap. Based on this information, the global planner calculates a trajectory between the start and goal positions [39]. The global planner does not take moving obstacles or robot kinematics into account. It only produces a high-level trajectory which should be refined by the local planner [39]. By default, Dijkstra’s algorithm [40] is used as a planning algorithm, although other planners such as A* [41] can also be selected [42].
- The local planner uses the trajectory from the global planner, the local costmap and the robot pose to calculate velocity commands. Contrary to the global planner, the local planner can take the kinematics and dynamics of the robot into account [39]. By using the local costmap, the planner also ensures that previously unknown obstacles are taken into account. By default, the ROS navigation stack uses the Dynamic Window Approach (DWA) [43] as a local planning algorithm.
- Finally, the robot controller sends actuator commands to the motors, based on the desired velocity returned by the local planner [44].

Together, these software packages enable a robot to localize itself, plan a path between its current pose and the desired goal, and execute that plan while avoiding obstacles. Besides sensor data, the navigation stack therefore requires a map of the environment, the initial robot position and a target position.

**Figure 5:**
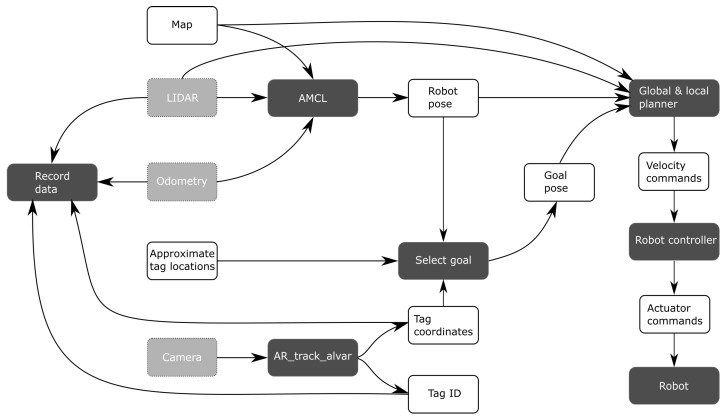
Overview of data flow during semi-autonomous calibration.

Semi-autonomous calibration assumes that the map of the environment as well as the approximate tag locations are known. At the start of the procedure, a human operator specifies the initial pose of the robot within the map. After this information is provided, the robot operates autonomously. The navigation stack then receives the nearest tag as its current goal. If a tag comes into the view of the camera while the robot is driving, it may deviate from that original goal. If the tag that is currently observed has not yet been visited, the robot will instead attempt to drive to the position directly underneath the tag. When it has reached that target, the robot stops moving and collects data for a few seconds. Next, the current tag is marked as visited, and the next goal is the closest nearby (unvisited) tag. When all tags have been marked as visited, the calibration is complete. Figure 5 shows a schematic overview of semi-autonomous calibration.

Semi-autonomous calibration thus requires some prior knowledge. However, high accuracy is not neccesarily required. The error on the initial location estimate can be reduced by AMCL as the robot gathers more information. The map will be optimized offline by cartographer. The map should only be accurate enough to enable the robot to navigate the environment. Not all obstacles need to be known beforehand, as the local costmap is updated continuously. Similarly, the tag coordinates do not need to be accurate, as the actual location will be measured during the calibration procedure. They are only used as goal locations for the robot to visit. Alternatively, if no tag locations are available prior to calibration, the user can specify a set of waypoints for the robot to follow. So long as the tags come into view of the camera at some point during the trajectory, they will be calibrated.

#### 3.2.3. Fully Autonomous Calibration

Fully autonomous calibration is similar to semi-autonomous calibration. The main differences can be found in the localization component, and the goal selection. Whereas semi-autonomous calibration uses a map and AMCL to localize the robot, fully autonomous calibration instead uses SLAM. Gmapping was used as a SLAM algorithm [45] during calibration, rather than the Cartographer SLAM algorithm that does the offline processing (see Figure 3). A different implementation was needed because the Cartographer software is not compatible with the exploration software that selects the robot goal poses. Gmapping has no offline optimizations; therefore, the robot location may be less accurate compared to Cartographer. However, this lower accuracy is not necessarily a problem, as long as the robot is still able to navigate autonomously.

**Figure 6:**
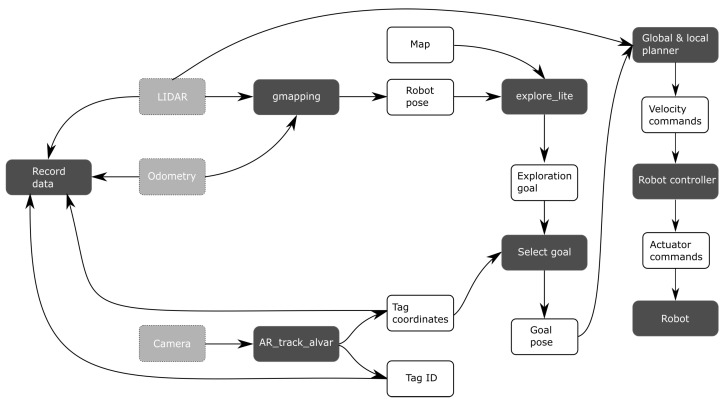
Overview of data flow during fully autonomous calibration.

Fully autonomous calibration obtains the target location from frontier exploration. The explore_lite software package is applied [46] for that purpose. Similar to semi-autonomous calibration, the goal selector will continuously check whether an AR tag is in view of the camera. If no tag can be found, the robot will continue to explore the space with frontier exploration. If a tag is visible and it has not yet been visited, the robot will deviate from its current goal and instead drive towards the AR tag. Again, the robot will attempt to position itself such that the AR tag is in the center of the image. Next, the robot stops moving and collects data for a few seconds, after which the tag is marked as visited. Frontier exploration then continues as usual, until either a new tag is observed or no more frontiers can be found. Figure 6 illustrated the fully autonomous calibration procedure.

**Figure 7:**
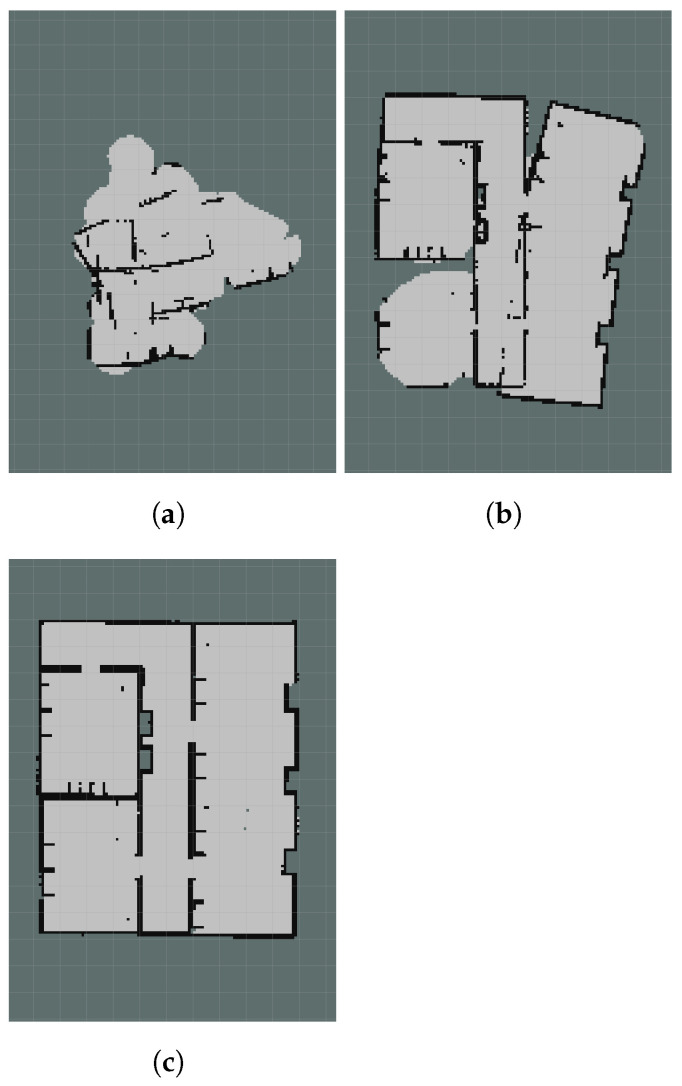
SLAM with artificially restricted LIDAR ranges. (**a**) Maximum range: 1 m. (**b**) Maximum range: 2 m. (**c**) Maximum range: 4 m.

Contrary to semi-autonomous calibration, fully autonomous calibration requires no prior knowledge. Consequently, some tags could be missed. If a tag never comes in the field of view of the camera, it will not be calibrated. This can be compensated to some extent by artificially limiting the range of the LIDAR data. By adjusting the Gmapping parameters, it is possible to exclude measurements that are far away from the robot. Therefore, the robot will have to drive further to explore all frontiers, and the likelihood of observing all tags is higher. However, limiting the range too much will negatively impact the SLAM accuracy, and might make exploration impossible. Figure 7 shows several maps built with different restrictions in the LIDAR measurement range. When the range is restricted to 1 m (Figure 7a), exploration fails entirely. Gmapping is unable to detect and match sufficient features in this small measurement range, therefore the map cannot be successfully generated during calibration. If the range is increased to 2 m (Figure 7b), exploration is occasionally successful, even though the map is relatively inaccurate. By increasing the range to 4 m (Figure 7c), mapping is more accurate even during online processing. Unless otherwise specified, the following sections will use a measurement range of 4 m.

### 3.3. Data Processing

For each of the three calibration approaches described in Section 3.2, 10 simulations are conducted. After the processing described in Section 3.2, each simulation provides a map of the environment with the tag locations. Ideally, we could directly compare the calibrated locations with the true positions. However, the results of the calibration are described in a different coordinate frame than the simulation environment. Therefore, we will determine the accuracy of the calibration by calculating the distance between each tag in both the map frame and the world frame:

(1) $$\begin{array}{cc} \varepsilon_{r,ij} & =\;\left|d_{sim,ij}-d_{est,ij}\right|\\ & =\;\left|\sqrt{{\left(x_{sim,i}-x_{sim,j}\right)}^{2}+{\left(y_{sim,i}-y_{sim,j}\right)}^{2}}-\sqrt{{\left(x_{est,i}-x_{est,j}\right)}^{2}+{\left(y_{est,i}-y_{est,j}\right)}^{2}}\right| \end{array}$$

where:

- $\varepsilon_{r,ij}$ is the error on the distance between lights *i* and *j*
- $d_{ij}$ is distance between tags *i* and *j*
- *x* and *y* indicate Cartesian coordinates.
- subscripts ${}_{sim}$ and ${}_{est}$ indicate the coordinates in the simulation environment and the coordinates as estimated by the calibration procedure, respectively.

These distances are independent of the origin and orientation of their coordinate frames; therefore, they can be used as a measure of accuracy. Every simulation returns several such distances. The average value of all distances from all simulations will be used as the main performance indicator. In addition, the 95th percentile of the cumulative error distribution will also be included as an indicator for the worst case scenario. This will be referred to as the P95 value.

Finally, besides the accuracy of a particular approach, the required calibration time will also be calculated by averaging the required times of each simulation.

## 4. Results

**Figure 8:**
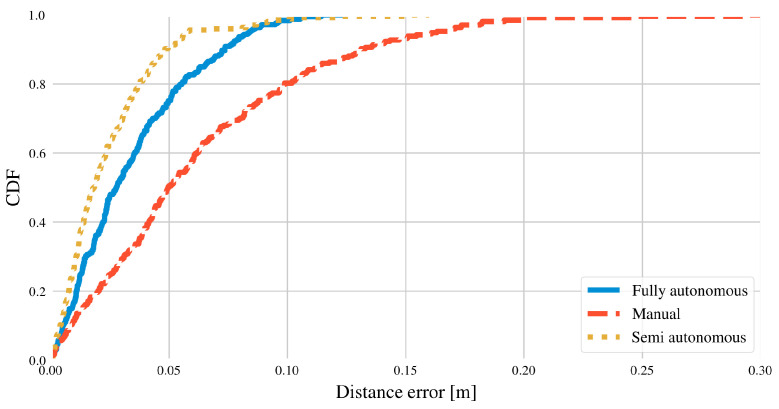
Cumulative error distribution for different calibration methods. The horizontal axis indicates the distance error as calculated by Equation (1).

Table 2 shows an overview of the main performance indicators for each calibration method. The values in Table 2 are obtained as the average value for all simulations. A complete overview of the error distribution can be found in Figure 8, which contains all error values from all simulations.

Table 2 and Figure 8 show that the automated calibration methods both perform significantly better than manual calibration. This observation can likely be attributed to the different methods for centering the tag during calibration. All calibration methods attempt to drive the robot directly under each light source and record data for a few seconds. However, manual calibration relies on the judgment of the operator, whereas the automated methods use an internal controller. The automated methods are able to better center the tag in the image. Those images will in turn have less distortion, and therefore the accuracy of the automated methods can be higher.

**Figure 9:**
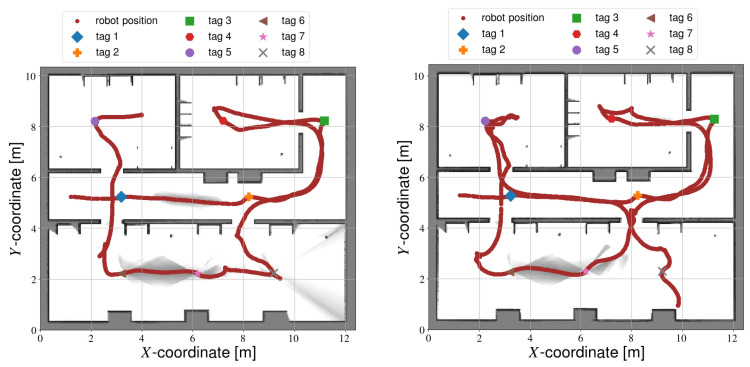
Calibration results from two different fully autonomous simulations. White pixels indicate free space, black pixels represent obstacles and grey areas indicate unexplored space.

**Figure 10:**
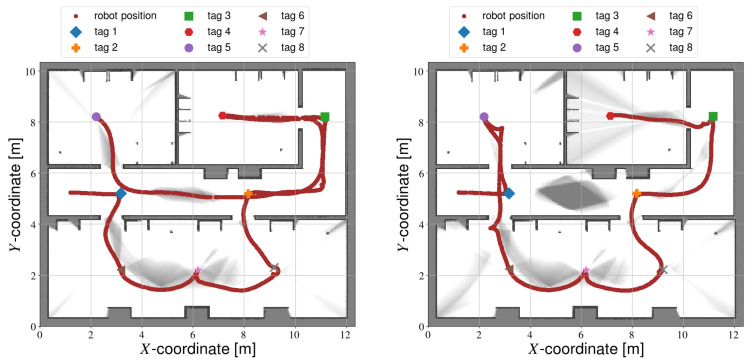
Calibration results from two different semi-autonomous simulations.

A smaller difference in accuracy can also be observed between the automated methods, as semi-autonomous calibration is slightly more accurate. This difference can likely be explained by the robot trajectory. During fully autonomous calibration (see Figure 9), the robot regularly takes sharp turns, and the overall trajectory is irregular. Consequently, the accuracy of the trajectory and map generated by the SLAM algorithm are negatively affected. In contrast, semi-autonomous calibration (see Figure 10) follows a much more regular trajectory, with smoother and more predictable motions. Hence, the accuracy of this method is slightly higher.

Both Figure 9 and Figure 10 contain some light grey areas. These are not necessarily unexplored sections, but rather areas of reduced probability. The Carthographer algorithm is somewhat different to other SLAM algorithms such as Gmapping in this regard. The map output is not binary, but rather a probability. As more information is gathered, the pixels become lighter or darker (corresponding to free space or obstacles respectively) depending on the observations. So in this case, the algorithm is less certain about these cells compared to the rest of the map. Additional observations would increase the uncertainty, but this can only be achieved by reducing the robot speed, or increasing the length of the trajectory. The right hand side of Figure 10 does have some unexplored areas, as the robot did not travel down the corridor in this case.

The error values for fully-autonomous calibration are also more spread out compared to semi-autonomous calibration. The trajectories followed by frontier exploration can vary significantly between experiments (see Figure 9). In contrast, semi-autonomous calibration largely follows the same trajectory every time (see Figure 10).

**Figure 11:**
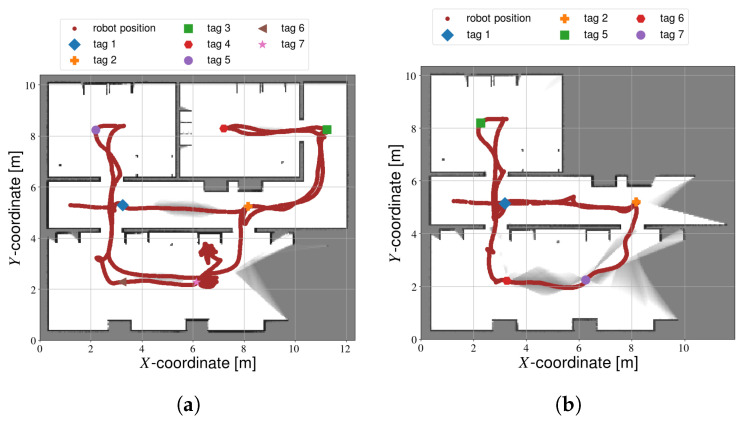
Calibration results from failed fully autonomous simulations. (**a**) Simulation where all but one tag was mapped. (**b**) Simulation that failed to map three tags.

It should be noted that not all of the fully autonomous calibration attempts were successful (see Figure 11). Out of the 10 simulations, the robot did not explore the entire space in two instances. In one of those cases, all but one tags were still calibrated (Figure 11a). In the other simulation, three of the tags were missed (Figure 11b). This is likely due to either an error in the configuration of the explore_lite package, or due to bugs in the software itself. The results from these two experiments were excluded from the timing results in Table 2. Exploration was not yet complete, so including these results would wrongly reduce the average trajectory time. Including or excluding the data had no effect on the average accuracy, however. The values of Table 2 do not change whether or not these simulations are included. For completeness sake, these data are still included in the compete CDF (Figure 8).

**Figure 12:**
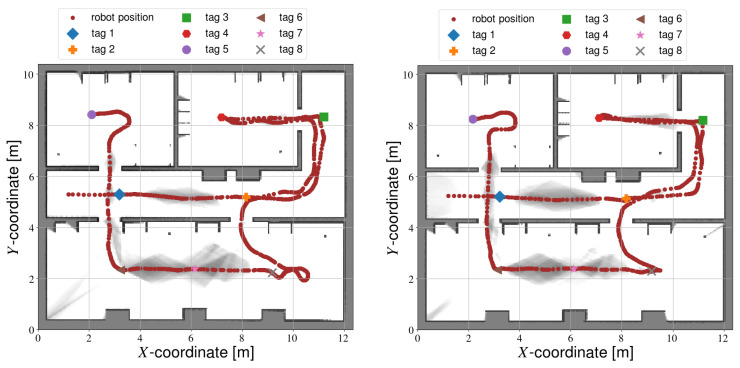
Manual calibration results.

Interestingly, the manual and fully automated calibration methods each take approximately the same amount of time to complete. While the overall trajectory followed by the autonomous robot is often longer, the movement speed is much higher compared to manual calibration. Semi-autonomous calibration follows approximately the same trajectory as manual calibration (see Figure 12), but is able to complete the data gathering much faster. On one hand, the robot velocity is higher overall. Additionally, centering the tag in the image can be done faster by the automated method. Therefore, semi-autonomous calibration is the fastest method.

## 5. Discussion

The calibration methods presented in this paper were evaluated in a simulation environment. Therefore, their relative performance is more important than the absolute error values. Additionally, the evaluation used in this paper replaced the lights with AR tags, similar to [31]. The focus of this work is not necessarily on the calibration itself. Instead, this paper is concerned with automating the data collection. Therefore, AR tags can be a suitable substitute for LEDs.

The results in Section 4 showed that semi-autonomous calibration is the best method overall, both in terms of accuracy and the required time. However, this approach does require a map of the environment and an approximate initial position of the robot at a minimum. A map could be obtained from building plans. If these plans are unavailable, one could use the robot to construct the map first. However in that case, manual steering would be required. Hence, it would be better to use then use those data for calibration directly, rather than first building a map and then calibrating. The initial position of the robot is to be selected by the user by clicking on the map. This initial estimate does not need to be very accurate, however. As the robot gathers more information, AMCL is able to improve upon this initial estimate. Ideally, the approximate light source locations would also be available for semi-autonomous calibration, yet they can be replaced by waypoints selected by the user (similar to how the initial position is specified). After this information is provided, no further human input is required.

Both manual and fully autonomous calibration require no prior knowledge. Both require a similar amount of time to complete, but autonomous calibration can be much more accurate. Additionally, a human operator is not required. Nevertheless, fully autonomous calibration does not always succeed with the current software setup.

Our simulations did not include any moving obstacles (such as people or other robots). In the case of manual calibration, it is up to the operator to decide how to approach them. The autonomous calibration methods both use DWA for obstacle avoidance. Therefore, they will initially respond the same way and attempt to go around the obstacle. However, in case the tag itself is blocked, the semi-autonomous calibration will still attempt to reach this position for some time. If it is unreachable, it will continue to the next tag and come back to the current one later. In contrast, fully autonomous calibration will look for another path the to frontier. Therefore, it may not observe the tag, and consequently not calibrate it.

In principle all the calibration methods discussed in Section 3.2 can be scaled up to multiple robots, but for some this is easier than others. In the case of manual calibration, surveyors need to coordinate with each other (e.g., each can visit a particular floor or section of a building). Semi-autonomous calibration can similarly divide the different goal locations (either the light locations or the way points) over multiple robots. Fully autonomous calibration is also possible with multiple robots. In that case, central coordination is needed, and the maps need to be merged online such that the robots know all visited areas and not just their own. The merging of maps can be achieved by packages such as multirobot_map_merge [46]. Alternatively, the rrt_exploration package can also use multiple robots at the same time [21].

The goal of a calibration procedure to determine the parameters required for positioning. Therefore, calibration is always offline process. In fingerprinting the calibration (that is, the construction of the fingerprinting database) is more explicit compared to range-based systems. However, both will need some form of calibration. The calibration methods evaluated in this paper can be used by range-based VLP systems. However, it would be relatively straightforward to adapt the procedure to the calibration of fingerprinting systems. Finally, it is worth noting that the calibration methods can be extended to the other positioning systems as well. For example, WIFI access points or RFID tags can be added to the map with an observation model similar to [17,47]. Alternatively, AR tags could be placed next to the signal transmitters at a fixed relative position. From the calibrated AR tag locations, the beacon location could then be calculated.

## 6. Conclusions

In this paper, we compared three different methods for calibrating a visible light positioning system: manual, semi-autonomous and fully autonomous calibration. Simulation results revealed semi-autonomous calibration as the most accurate and fastest method. Manual and fully autonomous calibration need approximately the same amount of time. Fully autonomous calibration is significantly more accurate, but does not always calibrate the entire environment.

Future work can further investigate performance of these approaches in the presence of moving or unknown obstacles. Additionally, calibration could be performed faster by using multiple robots simultaneously.

## Figures and Tables

**Table 1:** Main calibration parameters.

Parameter	Value	Unit
Common		
Marker size	5	cm
number of particles	0.1	s
Max new marker error	0.2	/
Max tracking error	0.2	/
Manual calibration		
number of particles	30	/
Semi-autonomous calibration		
planner frequency	20	Hz
min particles	500	/
max particles	3000	/
Calibration time per light	5	s
Max distance to light center	0.1	m
Fully autonomous calibration		
max LIDAR range	4	m
min frontier size	0.3	m
planner frequency	3	Hz
number of particles	30	/
Calibration time per light	5	s
Max distance to light center	0.1	m

**Table 2:** Overview of calibration results.

Calibration Method	Manual	Semi-Autonomous	Fully Autonomous
Average accuracy [m]	0.06	0.02	0.03
P95 accuracy [m]	0.16	0.06	0.08
Average trajectory time [s]	337.44	240.82	336.62

## Data Availability

The data presented in this study are available on request from the corresponding author.

## References

[1] Gezici S., Tian Z., Giannakis G.B., Kobayashi H., Molisch A.F., Poor H.V., Sahinoglu Z. (2005). Localization via ultra-wideband radios: A look at positioning aspects of future sensor networks. IEEE Signal Process. Mag..

[2] Zafari F., Gkelias A., Leung K.K. (2019). A Survey of Indoor Localization Systems and Technologies. IEEE Commun. Surv. Tutor..

[3] Liu F., Liu J., Yin Y., Wang W., Hu D., Chen P., Niu Q. (2020). Survey on WiFi-based indoor positioning techniques. IET Commun..

[4] Liu H., Darabi H., Banerjee P., Liu J. (2007). Survey of wireless indoor positioning techniques and systems. IEEE Trans. Syst. Man Cybern. Part C Appl. Rev..

[5] De Lausnay S., De Strycker L., Goemaere J.P., Nauwelaers B., Stevens N. A survey on multiple access Visible Light Positioning. Proceedings of the 2016 IEEE International Conference on Emerging Technologies and Innovative Business Practices for the Transformation of Societies, EmergiTech 2016.

[6] Do T.H., Yoo M. (2016). An in-depth survey of visible light communication based positioning systems. Sensors.

[7] Mendoza-Silva G.M., Torres-Sospedra J., Huerta J. (2019). A meta-review of indoor positioning systems. Sensors.

[8] Amsters R., Demeester E., Slaets P., Holm D., Joly J., Stevens N. Towards automated calibration of visible light positioning systems. Proceedings of the 2019 International Conference on Indoor Positioning and Indoor Navigation, IPIN 2019.

[9] Amsters R., Demeester E., Stevens N., Slaets P. (2021). Calibration of visible light positioning systems with a mobile robot. Sensors.

[10] Mautz R., Ochieng W. (2007). A Robust Indoor Positioning and Auto-Localisation Algorithm. J. Glob. Position. Syst..

[11] Kim Y., Chon Y., Cha H. (2012). Smartphone-based collaborative and autonomous radio fingerprinting. IEEE Trans. Syst. Man Cybern. Part C Appl. Rev..

[12] Rai A., Chintalapudi K.K., Padmanabhan V.N., Sen R. Zee: Zero-effort crowdsourcing for indoor localization. Proceedings of the Annual International Conference on Mobile Computing and Networking, Mobicom ’12, MOBICOM.

[13] Wang H., Sen S., Elgohary A., Farid M., Youssef M., Choudhury R.R. No need to war-drive: Unsupervised indoor localization. Proceedings of the 10th International Conference on Mobile Systems, Applications, and Services, MobiSys ’12.

[14] Yang Z., Wu C., Liu Y. Locating in fingerprint space: Wireless indoor localization with little human intervention. Proceedings of the Annual International Conference on Mobile Computing and Networking, Mobicom ’12, MOBICOM.

[15] Hähnel D., Burgard W., Fox D., Fishkin K., Philipose M. Mapping and localization with RFID technology. Proceedings of the IEEE International Conference on Robotics and Automation, 2004. Proceedings. ICRA ’04.

[16] Palaniappan R., Mirowski P., Ho T.K., Steck H., MacDonald M., Whiting P. Autonomous RF Surveying Robot for Indoor Localization and Tracking. Proceedings of the Indoor Positioning and Indoor Navigation (IPIN).

[17] Mirowski P., Palaniappan R., Ho T.K. Depth camera SLAM on a low-cost WiFi mapping robot. Proceedings of the 2012 IEEE Conference on Technologies for Practical Robot Applications, TePRA 2012.

[18] Lebreton J.M., Murad N., Lorion R. Real-time radio signal mapping using an autonomous robot. Proceedings of the 2015 IEEE Radio and Antenna Days of the Indian Ocean, RADIO 2015.

[19] Yamauchi B. Frontier-based approach for autonomous exploration. Proceedings of the IEEE International Symposium on Computational Intelligence in Robotics and Automation, CIRA.

[20] Le X.S., Fabresse L., Bouraqadi N., Lozenguez G., Chen Z., Mendes A., Yan Y., Chen S. (2018). Evaluation of out-of-the-box ROS 2D slams for autonomous exploration of unknown indoor environments. Lecture Notes in Computer Science (Including Subseries Lecture Notes in Artificial Intelligence and Lecture Notes in Bioinformatics).

[21] Umari H., Mukhopadhyay S. Autonomous robotic exploration based on multiple rapidly-exploring randomized trees. Proceedings of the 2017 IEEE/RSJ International Conference on Intelligent Robots and Systems (IROS).

[22] Alam F., Parr B., Mander S. (2019). Visible Light Positioning Based on Calibrated Propagation Model. IEEE Sens. Lett..

[23] Bastiaens S., Plets D., Martens L., Joseph W. Response Adaptive Modelling for Reducing the Storage and Computation of RSS-Based VLP. Proceedings of the IPIN 2018—9th International Conference on Indoor Positioning and Indoor Navigation.

[24] Rodríguez-Navarro D., Lázaro-Galilea J.L., Bravo-Muñoz I., Gardel-Vicente A., Tsirigotis G. (2016). Analysis and calibration of sources of electronic error in PSD sensor response. Sensors.

[25] Rodríguez-Navarro D., Lázaro-Galilea J.L., Gardel-Vicente A., Bravo-Muñoz I., De-La-Llana-Calvo Á. (2018). Indoor positioning system based on PSD sensor. Geographical and Fingerprinting Data for Positioning and Navigation Systems: Challenges, Experiences and Technology Roadmap.

[26] Bastiaens S., Raes W., Stevens N., Joseph W., Plets D. New Photodiode Responsivity Model for RSS-based VLP. Proceedings of the 2019 Global LIFI Congress, GLC 2019.

[27] Yue Y., Zhao X., Li Z. (2021). Enhanced and Facilitated Indoor Positioning by Visible-Light GraphSLAM Technique. IEEE Internet Things J..

[28] Liang Q., Liu M. (2020). An Automatic Site Survey Approach for Indoor Localization Using a Smartphone. IEEE Trans. Autom. Sci. Eng..

[29] Glass T., Alam F., Legg M., Noble F. (2021). Autonomous fingerprinting and large experimental data set for visible light positioning. Sensors.

[30] Koenig N., Howard A. Design and use paradigms for Gazebo, an open-source multi-robot simulator. Proceedings of the 2004 IEEE/RSJ International Conference on Intelligent Robots and Systems (IROS).

[31] Qin C., Zhan X. (2019). VLIP: Tightly Coupled Visible-Light/Inertial Positioning System to Cope with Intermittent Outage. IEEE Photonics Technol. Lett..

[32] Niekum S., Saito I.I. (2016). ar_track_alvar—ROS Wiki. http://wiki.ros.org/ar_track_alvar.

[33] Hess W., Kohler D., Rapp H., Andor D. Real-time loop closure in 2D LIDAR SLAM. Proceedings of the IEEE International Conference on Robotics and Automation.

[34] Marder-Eppstein E., Ferguson M., Lu D.V., Hoy A. (2016). Navigation–ROS Wiki. http://wiki.ros.org/navigation.

[35] Thrun S. (2002). Probabilistic Robotics.

[36] Macenski S. (2020). Navigation Concepts—Navigation 2 1.0.0 Documentation. https://navigation.ros.org/concepts/index.html.

[37] Marder-Eppstein E., Lu D.V., Hershberger D., Ferguson M., Hoy A. (2015). costmap_2d—ROS Wiki. http://wiki.ros.org/costmap_2d.

[38] Guimarães R.L., de Oliveira A.S., Fabro J.A., Becker T., Brenner V.A. (2016). ROS Navigation: Concepts and Tutorial. Stud. Comput. Intell..

[39] Marder-Eppstein E., Berger E., Foote T., Gerkey B., Konolige K. The office marathon: Robust navigation in an indoor office environment. Proceedings of the IEEE International Conference on Robotics and Automation.

[40] Dijkstra E.W. (1959). A note on two problems in connexion with graphs. Numer. Math..

[41] Hart P., Nilsson N., Raphael B. (1968). A Formal Basis for the Heuristic Determination of Minimum Cost Paths. IEEE Trans. Syst. Sci. Cybern..

[42] Lu D.V., Ferguson M., Hoy A. (2016). global_planner—ROS Wiki. http://wiki.ros.org/global_planner.

[43] Fox D., Burgard W., Thrun S. (1997). The dynamic window approach to collision avoidance. IEEE Robot. Autom. Mag..

[44] Marder-Eppstein E., Lu D.V., Ferguson M., Hoy A. (2016). move_base—ROS Wiki. http://wiki.ros.org/move_base.

[45] Grisetti G., Stachniss C., Burgard W. Improving grid-based SLAM with Rao-Blackwellized particle filters by adaptive proposals and selective resampling. Proceedings of the IEEE International Conference on Robotics and Automation.

[46] Hörner J. (2016). Map-Merging for Multi-Robot System. Bachelor’s Thesis.

[47] Milella A., Vanadia P., Cicirelli G., Distante A. RFID-based environment mapping for autonomous mobile robot applications. Proceedings of the IEEE/ASME International Conference on Advanced Intelligent Mechatronics.

